# Patterns of antipsychotic prescribing in first episode psychosis – differences between acute and early intervention services

**DOI:** 10.1192/bjo.2021.116

**Published:** 2021-06-18

**Authors:** James Fallon, Sophie McBrien, Keegan Curlewis

**Affiliations:** 1 Sussex Partnership NHS Trust; 2 Brighton and Sussex Medical School

## Abstract

**Aims:**

This study aimed to evaluate the patterns of antipsychotic prescribing in patients with first episode psychosis (FEP) at the time of their initial treatment and over the first year with the Early Intervention Service (EIS). It was hypothesised that different care teams would have a preference for certain antipsychotic medications and that initial medication choice would be continued through the first year.

**Background:**

Research indicates that with the exception of clozapine, all antipsychotics are equally as effective. However, anecdotally it has been observed that inpatient and crisis teams and EIS have differing initial medication choices.

**Method:**

An analysis of the North West Sussex EIS caseload (n = 67) was conducted. The first antipsychotic prescribed and initiating team was recorded. Prescribed medication for those that had completed 12 months (n = 43) with EIS after initial prescription was recorded. An analysis was performed of prescribing choice by initial care team (acute vs EIS vs other community services) with the frequency with which medication was changed during treatment.

**Result:**

97% (n = 65) of patients were started on an antipsychotic. Initial medication choice was olanzapine (44.8%, n = 30), aripiprazole (22.4%, n = 15), risperidone (20.9%, n = 14), quetiapine (6%, n = 4) and zuclopenthixol were least common (1.5%, n = 2). At the 12 month point 51.2% (n = 22 of 43) had switched and 16.3% (n = 7 of 43) had discontinued.

The most common medication started by acute services was olanzapine (56.0%, n = 28 of 50), though of those who completed 12 months this had been switched in 53% of cases (n = 9 of 17). EIS most commonly initiated aripiprazole or risperidone (37.5% each n = 4). At 6 and 12 month follow-up by EIS, the most commonly prescribed antipsychotic was aripiprazole (24 patients 40.7%, and 14 patients 32.6% respectively).

**Conclusion:**

There was a clear preference for olanzapine as initial treatment of First Episode of Psychosis in the region. On breakdown it was apparent that there was a split in prescribing choices between more sedating medication in acute services and less sedating medication in EIS. Given that most patients changed to less sedating and less metabolic active medications over their first year it is not clear why alternative options are not used at the start of treatment. Future research will focus on clinician's rationale for initial prescribing choice. This will look for any underlying bias toward specific medications.

